# Dr. Chisholm on Various Diseases

**Published:** 1822-09-01

**Authors:** 


### *A Manual of the Climate and Diseases of Tropical Countries, &c.* By Colin Chisholm, M.D. F.R.S. &c.

[Concluded from No. 9 page 121.]

In our first analytical article on the work before us, we concluded that portion of it which refers more particularly to the climate and diseases of equatorial regions, and reserved for the present article, those diseases, which (being more or less common to all climates,) are interesting to all classes of our readers. We did not touch, and shall not here touch upon, the third part of the work, because it is a condensed analysis of Dr. Chisholm's well-known Essay on the Malignant Pestilential Fever of the West Indies, denominated by the Doctor and several others, the Bulam fever. We are unwilling to revive disputes about names; but to the account of the malady itself, as drawn up most ably by our author, we refer our tropical brethren, leaving the doctrinal parts of the subject for their own consideration, when they have opportunities of witnessing that dreadful scourge in our transatlantic possessions.

We shall also pass over Cholera Morbus, because, although it is a disease sufficiently common in this country, our author treats of it only as it occurs between the tropics—especially in that fatal form denominated "spasmodic cholera," or "mort de chien." Dr. Chisholm's experience leads him to fully coincide with a late writer on the subject, that *bile*, so far from being a cause, is a salutary phenomenon in the disorder—"a copious flow of bile becomes a certain criterion of the cessation of disease." 85.

In a short chapter on colica pictonum, Dr. Chisholm informs us that, when he settled in the West Indies, 35 years ago, this disease was very common; but that now, it is hardly ever seen. He ascribes this change for the better, (and we think him right,) to the great change which has taken place, from intemperance to temperance, in the manners of the inhabitants. In respect to the disease in Devonshire, the operation of its cause was thus explained to him on the spot.

"In the summer and autumn, when the husbandmen are laboriously employed in the hay and corn harvest, the common practice of these men is to drink cider to the extent of their ability to buy, or rather, as it is allowed without limitation in hay harvest, to the extent of the capacity of their stomachs to contain it. The labour at this season produces an intolerable heat in their persons. Now the great cold of the cider, together with its harshness and acidity, acting against the heat produced by labour, gives rise to a spasmodic state of the bowels, which, acquiring its acmé in 24 hours, or even less time, in very many instances terminated in death. These labourers are so very inconsiderate, that, to allay the excessive heat and thirst occasioned by their work and the great heat of the season, they often drink to the extent of six or eight quarts of cider in the day; and, not unfrequently, such is their avidity and the uncomfortable state of their feelings, fill their stomachs at one draught." 94.

Dr. Chisholm has taken some pains to guard the young practitioner against confounding enteritis and colica pictonum—a mistake which, he thinks, would be fatal.

"The symptoms which distinguish colica pictonum from enteritis are these: the pain, at first, is rather more at the pit of the stomach; it afterwards fixes itself at the umbilicus, and thence darts in all directions over the abdominal viscera, accompanied by such retraction of the abdominal muscles as to oblige the patient to lean forward as the only posture in which he feels any thing like ease; whilst the circulation does not appear to be affected. In enteritis, the abdomen is tumid, prominent and hard, and the pulse is quick and full, the pain seems concentrated, and does not diverge in those spasmodic twitchings or dartings, observed in colica pictonum. In the latter, too, besides the rigidity and retraction of the muscles, the belly seems pressed or drawn towards the spine, with a force proportional to the degree of spasm. In enteritis there is no spasm. In colica pictonum there is soon perceived a disposition to paralysis in the extremities, and, often, a contraction of the joints; which in enteritis never takes place." 95.

Two modes of curing this disease are laid down by Dr. Chisholm—one by opiates and purgatives—the other by opium and calomel conjoined. "Either, if boldly adopted will effect the object in view."

In a very short chapter on chorea, Dr. Chisholm informs us that, in most cases, Dr. Hamilton's plan of purgatives totally failed; and indeed we have seldom seen chorea cured by purgatives. Latterly he cured six cases by blistering the sacrum. The patients were all young—from six to eighteen years old. The third application of blisters uniformly put a stop to the disease in all six cases.

The tenth chapter, on worms, contains many excellent observations. It is well known that, these animals make great havoc among the negroes of the West Indies, and, indeed, the children of the whites also. The great indigenous vermifuge of the West, is the spigelia anthelmia, or arabaca of Plumer. But its effects are so extremely violent, as to render its exhibition very unsafe. In Dr. Chisholm's European practice, nothing has been equal to the oil of turpentine, whether in tænia, teres, or ascarides. We shall quote one case from our author, exemplifying the good effects of ol. terebinth. in these complaints.

"The second case, was a lady at Clifton, about 25 years of age. From her symptoms, I suspected the presence of tænia;—an unsatiable appetite, constant and gnawing pain at the stomach, a wan complexion, melancholy state of mind, torpid bowels, &c.—I gave turpentine here, as a sure remedy to destroy tænia. She took six drachms unmixed. The consequence was truly surprizing. It acted in about an hour, and brought away, at one discharge, at least three pounds of solid fæces, not scybala, but impacted masses, and an amazing quantity of ascarides. The patient herself was terrified at an effect so unexpected, and, to her apprehension, so frightful—but my astonishment was greatly increased, when at the end of three days the dose was repeated, I found precisely the same result. Every symptom then ceased, and she got rapidly well.—Several cases have since occurred; and two of teres; all with equally happy results. Upon the whole, therefore, I am decidedly of opinion, that no vermifuge, hitherto employed, should supercede the use of oil of turpentine, which I have reason to believe will be always found a safe, a cheap, and most certain remedy, in every case of worms, whether tænia, teres, or ascarides." 100.

Dr. Chisholm takes this opportunity of mentioning his experience of oil of turpentine in several other complaints. He has exhibited this medicine with much benefit as an aperient in chronic hepatitis, and hepatic melancholia, or hypochondriasis. In many of these cases, oil of turpentine has more effectually cleared the bowels than calomel alone or combined with other deobstruents, and that without griping. This medicine seems to stimulate the whole line of the primæ viæ, to loosen the impacted fæces throughout all the convolutions, and make a speedy and effectual clearance. The occasional but short excitement of the urinary organs is the only inconvenience Dr. C. has experienced from the oil.

On the chapter embracing pneumonia we shall not dwell. Dr. Chisholm, after a copious detraction of blood, at the beginning of the disease, trusts rather to nitre, calomel, and antimonial powder, taken every three hours or oftener, than to repetitions of the bleeding and digitalis. This powder should be assiduously continued till the constitutional effects of the mercury are evident, when the pulmonic symptoms will give way. This is a modification of Dr. Hamilton's well known plan of treatment; but whether it is an improvement, our own experience does not enable us to judge. Our author seems to lament the havoc which is made by depending on bleeding as long as the blood exhibits the buffy coat, "and digitalis until the system labours under what has been called morbus digitalis." He has observed this practice, "so often followed by phthisis and a lingering death, that it should be discarded from the physician's mind." For our own parts, we never depend on any such criterion as the coagulable lymph of the blood. We look entirely to the functions of the lungs and heart—till they are free, we bleed whether there be buff or not; and when they are free we desist, even were the last cup a mass of inflammatory crust.

In respect to phthisis, our author truly observes that it is not by low latitude or approximation to the equator, that we are to calculate on paucity of this dire scourge of Europe:—"it is the peculiar localities of places and countries, and the degree of exposure to cold northerly winds, &c." which constitute the liability to pulmonary inflammation, and, of course, to phthisis. This disease as certainly follows pulmonary abscess between the tropics, as in England; but it runs its course much quicker. The sensation of cold, and its effects on the human system, are relative in all climates:—thus the cold of 65° or 70° has the same effect in the torrid zone, if suddenly applied while the body is heated by exertion, or enveloped in the usual sun-heat of 100 or 120, as that of 32° in northern countries, under similar circumstances. The observations of our friend Dr. Clark on this subject are peculiarly valuable, and are applicable to the point under consideration. As far, however, as regards the predisposing cause of phthisis, viz. scrophula, a tropical climate undoubtedly is unfavourable to its production. Scrophula, in fact, is nearly unknown between the tropics.

"Within the tropics," says our author, "but more especially in the West India islands, we have changes of temperature and perflation in turning round every projection, and in receding into every hollow; profuse perspiration and corrugation and dryness of the skin succeed each other, as these projections and hollows do. If, therefore, means are not resorted to, to provide against these quick changes, the sympathy between the skin and the lungs, will be forcibly excited, and inflammation and congestion in the latter will follow. Pneumonia and phthisis will be, as certainly, (perhaps more certainly,) the consequence, as in a cold climate, where such quick succession of inequality of surface, and such quick alternation of heat and cold, are not so frequent. I have already pointed out (part 1. ch. i. and iv.) how much the agency of this singular versatility contributes to the production of disease, and more especially of pulmonary and hepatic inflammation in the West India islands. It is, indeed, a most prolific cause, when not counteracted by temperance, and a care to preserve the skin from its peculiar and pernicious effect. I may venture, therefore, to say, that pneumonia and phthisis, as far as temperature and the vicissitudes of it are concerned, are, in proportion to the population at least, as frequent within the tropics, as in cold and temperate climates." 110.

In the Mediterranean and West Indies this may be the case; but we do not think that the East India climate comes under the same rule. Dr. Chisholm seems to view the incipient stage of phthisis as decidedly an inflammatory one, and that consequently there is nothing to be depended on, in this stage, but the antiphlogistic treatment. Whether it is strictly an inflammatory process by which the tubercle itself breaks down into a fluid resembling matter, is doubtful; but there can be no doubt that the cellular substance surrounding the tubercle is disorganized by inflammatory action, in the purulent stage of the disease, and kept in a state of inflammatory irritation during the enlargement of the tubercle, or incipient stage of phthisis. This view of the subject leads, of course, to antiphlogistic measures and counter-irritation, bearing in mind the scrophulous constitution with which we have to deal, and which certainly requires caution when depleting measures are pursued.

It is a curious fact, that *all changes* of climate, even from a warm to a cold one, are serviceable for a time to the phthisical invalid. We cannot resist the temptation to insert the following extract from our intelligent and experienced author.

"When purulent expectoration, laborious respiration, pain in the left side, emaciation, hectic flushings, and other symptoms of impending, or actually formed phthisis, are observed, there is no safety by remaining in the climate. It must be immediately changed, or measures must be adopted which, in their effect, may in some degree be equivalent, otherwise death is inevitable. A sea-voyage and a temperate or cold climate, present the only, or at least, the best chance of life. Medicine in this case, with a view to cure, is totally useless; and only tends to raise hopes which never can be realized, or to lull the unfortunate patient into a fatal security. If a voyage to any part of North America, Great Britain or Ireland, or to Bermudas, cannot be accomplished, from deficiency of means to defray the expence, the next best measure will be to cruise among the islands. To remain stationary is to wait the certain approach of death. The instances of the benefit derived from frequent change of climate are many. I have known life preserved and rendered comfortable for many years, by a plan of this kind. I shall mention only one instance—a gentleman who had often been my patient in Grenada, adopted it with complete success. His phthisical constitution was formed, within the tropics in early life, by neglect and improper treatment of pulmonary inflammation.—He possessed the power of change, and was relieved in North America.—But after a few months residence there, the symptoms began again to develope, and he returned to Grenada. He was, again, apparently restored to health; but after two years residence, he became again unwell; and now changed to his native country, Scotland, where he recovered and remained some time. But his complaint then returned, and for relief he resorted to the tropics. In about six years, by thus constantly changing the climate, on the re-appearance of the symptoms of phthisis, this gentleman had his health perfectly well established; as a security, however, and being possessed of ample fortune, he continued to make a frequent interchange of climate. The fatal insurrection in Grenada in the year 1795, was, at length, the cause of his death; for having made great exertions in the cruel warfare consequent upon that calamitous event, which his constitution was not equal to, he sunk under them, after more than twenty years of well established health," 111.

Our author has seen some remarkable instances, where an active bustling occupation, with temporary exposure to what may be deemed hardships, such as often occur in military or maritime service, during active campaigns or expeditions, have produced a wonderful change in constitutions apparently ruined by phthisis. "One thing," says our author, "is most certain, that confinement to the atmosphere of a room, or even house, is most highly prejudicial; it renders the person infinitely more susceptible of the impression of cold, and thereby tends to augment the evil which it is supposed calculated to remedy."

"A method of cure of phthisis pulmonalis, different in the means, but not dissimilar in principle, to that mentioned in the last paragraph, has been for some time adopted, with very considerable success.—In one case, a youth of eighteen, a near relative of my own, the practice was used, certainly with great benefit.—The practice employed in this case, was begun by sponging the chest and arms with a mixture of one part of vinegar and two of water, made moderately warm; this was immediately followed by dry friction with a flesh brush, or a piece of flannel. After a short time this was done to the whole body, that is, the sponging and dry friction daily, gradually reducing the mixture to coldness.—When this was done so long as to produce an evident change for the better, in the symptoms, the vinegar was used alone at night, and cold water in the morning. The diet was nourishing, but simple, and consisted in equal but small proportions of animal and vegetable food.—A little wine diluted with water at first, was allowed, afterwards it was allowed plain. The only medicine given was the following. Two ounces of bark were boiled in a quart of water for ten minutes—it was strained, and two ounces of the tincture of bark, and a pint of port wine were then added. Of this a wine-glass full was taken every day.—Moderate and amusing exercise was enjoyed in the open air.—This plan adopted with great benefit in the case of my relative is pretty nearly the same as Dr. Stewart's M. D. minister of Bolton, as described in a letter, I believe, from himself, and published by Dr. Sutton. (See Edin. Med. and Surg. Journ. vol. ix. p. 356.)" 113.

Before concluding the subject of phthisis, our author begs leave to recommend "the inhalation of the effluvia from raw Muscovado sugar." He has known it produce a wonderfully soothing effect, "which has, in some instances, become permanent." In Europe, a small barrel, or even basin, filled with the coarsest and dampest Muscovado sugar, may be placed in the corner of the room occupied by the patient. But in the West Indies it would be better to lodge the invalid near the boiling house of a sugar plantation—"and in changing the climate, a sugar-laden ship is the best conveyance."

Our author, after drawing a melancholy but a faithful portrait of that wretched vicious indulgence between the tropics, and indeed every where else, namely, grog-drinking and other species of inebriety, remarks that, when the mischievous and degrading habit happens to be subdued by right reasoning and acting, there generally remains a pain or almost indefinable sensation at the stomach, which is very distressing, and proves a great temptation to have recourse to the original cause of the evil.

"The remedy I have long and successfully advised for this, is a very simple one, a moderately sized tumbler of water, as warm as the patient can conveniently take it, on getting out of bed and dressing in the morning. This simple remedy has more efficacy than any other I ever tried;—it sooths the stomach, disposes the bowels to discharge their contents, encourages the secretion of bile and other fluids employed in digestion, occasions a warm glow on the surface, throws out a gentle perspiration, and finally gives a desire for breakfast.—If there is acid eructation, a small quantity of the infusion of camomile flowers, with magnesia, in the proportion of a small tea-spoon full of the latter to two or three wine-glasses full of the former, will be found a pleasant combination of a tonic and absorbent. Should the acidity prevail, and be attended with pyrosis, lime water is proper; but the most likely to allay these symptoms is the oxyd of bismuth rubbed up with a little sugar and powder of cinnamon. The dose should not exceed four grains of the oxyd thrice in the day, at least that is the quantity I have found most beneficial to adults. I make use of the word allay, because, as all these symptoms, generally, if not, always, depend on, and should be considered as indicating hepatic derangement, so should those means be resorted to, which mainly apply to the removal of that derangement. Thus, therefore, those absorbents should be employed as accessories, and mercury given, in the manner stated under chronic hepatitis as the principal agent. The oxygenated muriate of potash has done much good in chronic hepatitis brought on by habitual ebriety, or dram and grog drinking. As a tonic when those horrible feelings I have described, have yielded to moral and physical regimen and medical treatment, the following pill has answered better than any thing I have ever tried. ℞. extract gentian. ℈i. extract. colocynth. comp. gr. x, antim. tart. gr. i. ℳ optimè et divide in pilulas octo—sumat unam bis terve in die." 116.

We shall here take notice of the appendix to the work before us, which contains some observations on the climate of Geneva, and the relative prevalence of phthisis there. It appears that very accurate registers are kept at Geneva by a medical officer appointed for that special purpose. The population of that place is 24,000, and the deaths one in fifty annually. This is almost exactly the same as in England. But then the deaths by phthisis at Geneva, and in England are very different. In the latter country it is one in 224 of the population annually—in Geneva it is only one in 521. It appears also from the tables, that the mortality occurs chiefly at ages below 15 and above 40.

"These results are the more extraordinary, when we consider the peculiar localities, and their effect on the climate of Geneva. Geneva is placed nearly in the gorge of a long and broad valley, containing some of the most beautiful scenery in the world, its fine, blue, transparent lake, and a considerable portion of two very rapid rivers, the Rhone and the Arve;—confined by the nearer and lower ridges of the Savoy Alps on one side, and by the Juras on the other; —and terminated by very narrow passes through lofty mountains at the Ecluse on the SW. and at Villeneuve on the NE. It is thus necessarily subject to strong currents of wind from the SW. and NE.—and no other points, which are sometimes frightfully furious; —the former generally bearing on its wings watery clouds and heat; the latter excessive dryness, no clouds, but often piercing cold. From this peculiarity of situation, the climate of Geneva possesses sometimes and in some years, in winter, almost the cold of Russia, and almost the heat of South Italy in summer. Farenheit's thermometer in the shade and exposed to the north, was as high as 92° in August, and as low as 5° in January 1820. This, however, was a year of extraordinary heat and cold—and in general the mean temperature of the year is pretty nearly that of the West of England, being 55°.—The city of Geneva is built on the sides and summits of two hills of small elevation, on each side of the Rhone, where it issues from the Lake, giving it the aspect of remarkable beauty, and singular irregularity in its outline—but its streets are very narrow, the houses very high, built of hewn stone chiefly, each having a common stair-case, and every story or floor constituting a distinct house, or suit of apartments for a family.—The city is consequently subject to a variety of cross currents of air, and in many parts to a complete abstraction from the rays of the sun: so that on entering the city from the country a person experiences, in an instant, a very considerable change of temperature. In consequence of this arrangement too, Rheumatism and catarrhal affections are very common; and perhaps, to this sudden alternation of cold and heat, may be attributed, indirectly, that females are so subject to the goitre or bronchocele; their mode of dress, which leaves the neck exposed at all seasons and all temperatures, giving great effect, in that part, to the action of those sudden changes, and these cross currents of air." P. 335.

These particulars may be interesting to the British profession, as Geneva is now the residence of very many English families—and as all travellers pass through this celebrated city on their way to "Latium's velvet grounds."

We are reluctantly compelled to pass over the sections on coup de soleil, phrenitis, hydrocephalus, rheumatism, ophthalmia, and other inflammations, as we perceive that our limits will be overstepped. These sections contain many highly valuable observations, which well deserve the tropical reader's attentive perusal.

The 15th chapter, which is a very long one, embraces an extensive review of the history and treatment of tetanus, which is a severe scourge between the tropics, and too often baffles the powers of medicine in Europe. We are sorry to say that no satisfactory conclusion results from our able author's learned researches. His own experience leads him to trust more to *depletion and mercury*, as the *principal* agents in the methodus medendi, than to any other plan which has yet been proposed or acted on.

The same remark may be made on Dr. Chisholm's section on hydrophobia—a still more indomitable disease than tetanus. Our author, however, has brought forward into a focus all that is known of the pathology and treatment of this direst of all afflictions, and left his readers to judge for themselves, as to the most eligible plan to pursue. His own judgment inclines him to venesection and mercurial ptyalism, as the surest means of checking the hydrophobic inflammation.

From the concluding part of this chapter, in which some cases and observations are introduced on the subject of catalepsy, and cataleptic, maniacal, and hepatic hysteria, we shall make a short extract.

"These diseases seldom, indeed, occur within the tropics: nor should I have introduced them into a work confined to the history and treatment of tropical diseases, had I not experienced the beneficial effects of the depletory and mercurial treatment of them since my return to England; and had they not thereby furnished additional illustration of the propriety of that treatment, in the two important diseases which constitute the subject of the two preceding sections of this chapter. Catalepsy and cataleptic, maniacal, and hepatic hysteria, have been cured by a similar but much less rigid process. Only one case of the first, six of cataleptic maniacal, two of maniacal, and, at least, forty of hepatic hysteria, having been cured by moderate bleeding and purging in the first instance, and afterwards by mercurial ptyalism. These diseases, which there is every reason to believe depend on a similar but less intense inflammatory diathesis, and a similar locality of that diathesis, as that observed in tetanus and hydrophobia, are arrested in their progress by these means, and the cure has been completed by mild tonics combined with anti-spasmodics, in the following form. ℞. oxyd. zinci, gr. $\frac{1}{4}$ ad gr. iss, assafœtidæ—extract. rad. valerian.—Hyosciami āā gr. iss. ℳ. ft. pilulæ duæ pro dosi sumendæ 3 vel 4 in die." 157.

Some very interesting cases are introduced in illustration, for which we must refer to the work itself.

The volume concludes, as we before stated, with an excellent abridgement of the author's former work on the pestilential fever, commonly (but he thinks erroneously) called the yellow fever. Whatever discrepancies of opinion may exist as to the etiology and nature of the disease, all must allow that Dr. Chisholm has handed down to posterity a very valuable history and description of the disease and its treatment—a series of *facts* which must stand, however *doctrines* may revolve, rise, and sink again. This part of the work, therefore, will prove an important document in the hands of the young tropical practitioner, and to his careful perusal we recommend it.

Having fully expressed our opinion, in a former article, of the merits of the work, and of the character and talents of the author, we need not here repeat that opinion; but we cannot avoid introducing Dr. Chisholm's pious, zealous, and affecting farewell to his professional brethren, before we close the book.

" Having now finished what I proposed to offer for the guidance and instruction of the young and unexperienced in the diseases of tropical climates;—and having with this manual, for ever quitted the practice of my profession, I affectionately take leave of them, and of my medical brethren in general, assuring them of my most sincere respect, and of my most cordial wishes for their happiness and prosperity; and wishing them to be persuaded, that, if ever any remarks, or any language of mine, have given pain, or offence; if ever I have betrayed animosity in the maintainance of my own, or asperity in the consideration of another's opinion;—fully aware of the absurdity of a conduct, which the frailty of human nature, and the limited capacity and knowledge of man, can give no sanction to; I here solemnly abjure, and entreat pardon for. And now I most earnestly and fervently implore the true, the Almighty Physician to shed the influence of his blessed Spirit on these my labours; most humbly and devoutly trusting that, should it please Him to make me, thus, an instrument, by which a ray of light may be thrown on the dark path of Tropical Pathology, He will be graciously pleased to render that light more vivid, and those minds it is intended to illumine, still more open to receive its impression; so that a more clear perception of the obstacles, difficulties, and dangers, they have to encounter in their road, may be established; thereby giving that road more smoothness, more safety, and more simplicity, in conducting them to the grand object of our united efforts, the preservation of health, and the cure of disease, in a country where the former has been uncertain, and the latter too often impossible, under existing circumstances." 233.

*Geneva, May* 1821.

Amen! say we. And in the name of the profession (for we are sure our readers will silently but sincerely unite with us) we proffer to our venerable brother in retirement, the homage of reciprocal respect, esteem, and fervent wishes for his happiness. May he experience, amid the romantic vallies and majestic scenery of Switzerland,—

" What nothing earthly gives or can destroy,
" The soul's calm sunshine, and the heartfelt joy;"

resulting from the retrospect of a well-spent life here, and a confident anticipation of the joys of a world to come.

